# Black adolescents’ motivation to resist the false dichotomy between mathematics achievement and racial identity

**DOI:** 10.1038/s41539-024-00219-9

**Published:** 2024-03-02

**Authors:** Melody Wilson, Jamaal Sharif Matthews

**Affiliations:** Marsal School of Education, University of Michigan, Ann Arbor, MI USA

**Keywords:** Education, Psychology

## Abstract

This study investigates the racial-mathematical identity profiles of Black American adolescents. Survey data were collected in five schools across one U.S. urban school district at two time points (spring 2018 [*n* = 197] and spring 2019 [*n* = 210]). Based on extant research regarding psychological response patterns to racialized school stress, we investigated the existence of an identity negotiation pattern in which students were motivated to resist negative stereotypes about Black people by achieving well in mathematics. We conducted a latent profile analysis combining students’ self-beliefs across five indicators: racial centrality, racial public regard, mathematics attainment value, mathematics mastery experiences, and resistance motivation. Three distinct racial-mathematical identity profiles emerged: (1) Mathematics Devalued/Ambivalent, (2) Moderately Math Attained, and (3) Resistors. We found associations between profile membership and students’ gender, negative math emotions, and their receipt of cultural and critical mathematics instruction. We also found an association between the identity profiles and school type (academically selective “magnet” schools vs. open-enrollment neighborhood schools), but not in the direction that might be assumed. Moreover, we found that certain school environment factors (i.e., racial stereotyping and cultural and critical mathematics instruction) were significantly different in racially diverse magnet schools than in the neighborhood schools. Overall, our data reveal the existence of a highly motivated Resistor profile among Black students, that is predicted by cultural and critical mathematics instruction but underrepresented within this district’s selective magnet schools.

## Introduction

[My parents] They would tell me, “When you look in the mirror, you’re Black! You cannot change that,” … Like it’s not a bad thing being Black, it’s not. That’s one of the best things to be in life. Like it’s good, but you realize that you have to work ten times harder, because people is judging you off of what they think like a stereotype or a statistic… I don’t want to have an excuse. I want to be like, “Math wasn’t my subject at some point of time, but I pushed myself, and I was able to overcome that.

- Shayla, ninth grade Algebra student^1^

For many Black American students, early-to-middle adolescence represents a unique developmental stage where they are fostering a sense of their racial identity while also negotiating their academic selves, particularly within secondary mathematics classrooms. Beginning around sixth grade (~11–12 years old), the rigor, complexity, and abstract density of mathematics begins to increase considerably; regardless of race, many students experience decreased motivation and increased emotional cost in mathematics by the end of eighth grade^2^. At the same time, the development of social consciousness and recursive perspective-taking during adolescence allows youth to perceive both the stigma associated with their group (e.g., Black Americans as anti-intellectual and underachieving^3,4^) as well as the social prestige associated with certain scholastic abilities (e.g., mathematics aptitude as an indicator of intellectual giftedness^4^). Thus, for historically marginalized students the burden of managing the increasing challenges of secondary mathematics alongside navigating racial stereotypes and stereotype threat may exact additional psychological and emotional costs. The current study uses latent profile analysis (LPA) to explore patterns in how Black American adolescents negotiate their racial identity and mathematical value in concert. We then model associations between these identity negotiations (i.e., the profiles), school-related variables, mathematics pedagogical patterns, and students’ sense of emotional cost for engaging mathematics.

Expectancy-value theory^5^ (EVT) suggests the degree to which students value mathematics and expect success in it (e.g., math self-efficacy) predicts their engagement, persistence, and achievement in math. However, this perspective may mask the complexity of racially minoritized students’ experiences of stereotypes, stigmas, and biased curricula, all of which can affect their self-beliefs in mathematics. These challenges are evident in Shayla’s quote, which underscores racial identity and EVT constructs simultaneously. She expresses her racial centrality (i.e., the importance of being Black^6^) and public regard (i.e., awareness of society’s perception of Black people^6^) within the context of evaluating her mathematical effort and mindset. Further, she not only conveys strong math self-efficacy (i.e., the belief that she can work hard and become successful in math^7^), but also demonstrates a resistance-motivation mindset (i.e., the desire to defy stigma through academic achievement^8,9^). However, her recognition of having to “work ten times harder” underscores the cost of her mathematics engagement and persistence. More recently, EVT scholars have included cost (e.g., effort, time, and emotional cost) as a third major factor in this theory of motivation^2,10^. However, “racial opportunity cost”—the psychological, community, and representational costs of belonging to a racially minoritized group within a dominant-normed academic environment^11,12^, remains underdeveloped conceptually and underexplored empirically.

Altogether, Shayla’s quote complicates EVT and synopsizes a growing body of research on how Black American adolescents’ racial identity development relates to their scholastic outcomes. For example, racial centrality has proved to predict academic performance, valuing school, and academic self-concept for Black American youth^7–9^, and typically stems from parents’ racial socialization messages^13,14^. However, as adolescents mature, school-based factors begin to increase as socializing influencers^15^, which warrants continued research on the role of school racial climate for understanding adolescents’ racial identity development as well as their motivation in mathematics. For Black-American students, racial stereotype threat has been shown to affect both expectancy and value in STEM (science, technology, engineering, and mathematics)^16^.

Throughout the history of U.S. education, schools have socialized children around race both institutionally (e.g., segregation, tracking, curricular bias) as well as interpersonally (e.g., stereotypes, teacher and peer bias). Although de jure exclusion from “White” schools was a hallmark of the Black American schooling experience before the 1954 Brown vs. Board of Education decision, school segregation in the U.S. still persists today due to residential de facto segregation of Black families^17^. Despite its evolution over time, school segregation has ultimately resulted in unequal school resources and opportunities for Black children^18^. However, during the desegregation movement, Black parents were aptly concerned about the psychological and emotional costs their children would face as a result of discrimination within newly integrated schools^19^.

The fear of discriminatory (and thus psychological) challenges facing Black American youth during school integration were largely realized, especially through structural and social mechanisms such as tracking^20–23^, pervasive stereotyping, and biased low-rigor curricula. Racial stereotyping, in particular, is broadly known for its pernicious effects on the academic performance and psychological well-being of students of color^24,25^. Despite ample research evidence to the contrary^26^, Black American students are typically characterized as “caring less about school” than students from other racial groups^27,28^. In mathematics in particular, students become aware of racial stereotypes in elementary school and begin endorsing these beliefs during their secondary adolescent years^29^. Ultimately, those racial stereotypes begin impact their expectancies for success and value of mathematics over time^16^.

Both school segregation and racial discrimination within integrated schools have implicitly socialized Black adolescents around their racial identity. An emerging awareness of racial identity (with its accompanying stereotypes) necessitates the development of coping strategies–internal and external responses to the racial stigma constantly confronting Black youth. Some adolescents may respond by distancing themselves from their racial identity (self-devaluation^30,31^) from academics (academic devaluation^32–34^), or from both. The psychological consequences of racial discrimination in schools were foreseen by Black historian and educator Carter G. Woodson^35^ early in the twentieth century: “If you make a man feel that he is inferior, you do not have to compel him to accept an inferior status, for he will seek it himself” (p. 40). Woodson’s prescient statement encapsulates both self-devaluation and academic devaluation responses to discrimination. Further, empirical research has shown some evidence of these response patterns across both predominantly White and predominantly Black schools^36–39^.

However, in contrast to self-devaluation or academic devaluation, Woodson discussed a third response: resisting oppression by encouraging the Black community to value its own intellectual contributions to the world. This third response has also been observed in contemporary Black youth: a resistance motivation against pejorative racial ideologies through a persistent pursuit of academic success. O’Connor^40^ found that, despite an awareness of institutional discrimination and structural barriers, a group of high-achieving Black American students did not develop pessimistic dispositions toward their future success, decrease their effort expenditure, nor unhinge themselves from their Blackness. Rather, their unique knowledge of the legacy of Black struggle throughout U.S. history, as well as their connections with successful Black role models, helped them leverage the power of collective Black action to “come together” and “fight back” against racial subjugation^40^. Studying this resistance response may be of utmost importance given how little we know of it from an empirical basis.

Resistance motivation has typically been studied through small and primarily qualitative samples yet remains relatively unstudied in quantitative research. By examining a larger group of participants, quantitative modeling may identify more generalizable trends and patterns within this population, which may in turn generate new questions for both qualitative and quantitative researchers. Also underdeveloped in this body of literature is an integrated framework that underscores the school and classroom factors that predict various psychological negotiation patterns (i.e., are these patterns products of individual choice, or are they predictable by school and classroom characteristics?) The current study aims to address these gaps by exploring how differing school types, and the racial stereotyping therein, predict resistance motivation and other diverse negotiation patterns in mathematics classrooms.

One pathway many urban school districts have taken to address segregation and inequity in U.S. public education has been the creation of publicly funded “magnet” schools with specialized emphases such as language immersion, STEM-intensive programming, or the arts. At the secondary level (middle and high school), these schools are often academically selective, lending them special prestige. Designed to attract White students to schools located in predominantly Black neighborhoods^41^, magnet schools tend to have a greater racial-ethnic diversity among students compared to more segregated “neighborhood” schools, fostering an appearance of good-faith efforts to follow federal diversity guidelines^42,43^.

However, studies focusing on within-school segregation have exposed concerning problems in magnet schools. In a nationally representative sample Davis^44^ found that magnet school status was not associated with any decrease in White-Black segregation across classrooms and academic tracks. Furthermore, in a large survey of clustered inter-district magnet schools^45^, Black students reported significantly lower quantity and quality intergroup relations than White students. Several qualitative studies have further illustrated the emotional and psychological cost^11,12^ of Black students’ experiences of racial isolation and academic hierarchy within magnet schools, especially in STEM subjects^20,46–48^. In a retrospective case study on her own magnet school^47^, Gersti-Pepin stated, “The school was ~32% White and 68% Black. All my college preparatory classes were filled with white students. The two or three Black students were from predominantly middle-class white neighborhoods on the west side of the city” (p. 50).

School districts tout magnet schools’ academic excellence^49^, and Black parents have fought for their children to gain admission to these schools^50^. However, to date, no study has evaluated the effects of secondary magnet school ecologies on Black students’ psychological outcomes. If Black students are enduring racialized and emotional costs in the very schools that purport to address racial inequity, this might be associated with their response patterns to racial socialization and, ultimately, their academic performance. Black students who face daily prejudice in racially integrated magnet schools may not have adequate support to make sense of and navigate this reality. The current study begins to address this question by comparing Black students’ mathematics- and race-related psychological responses across one U.S. city’s magnet and neighborhood schools.

Alongside the institutional responses to racial inequality in education (e.g., magnet schools), other approaches have focused on interpersonal responses (e.g., classroom pedagogy, teacher cultural competence). Stemming from the U.S. civil rights movement of the 1950s–1960s led by Black Americans, the multicultural education movement sought to develop teachers’ cultural sensitivity, teach youth about their ethnic-racial heritage, integrate culture into curricula, and increase achievement and equity for students of color^51,52^. Through these mechanisms, multicultural education has predicted increases in learning and engagement for all students (not only students of color) and healthier intergroup relations between students of different ethnicities^53^. In particular, positive internalized race consciousness has been shown to correlate with achievement for Black students^54^.

However, over time, much of multicultural education has become reduced to merely “celebrating diversity” or “promoting tolerance”. In response, more “critical” pedagogies have emerged that explicitly address race and power. Critical multicultural education (sometimes known as multicultural social justice education) has emphasized identifying and examining inequality in society and prepares students to critique and act against social injustice^55,56^. Core to critical multicultural education is leveraging the connections between students’ cultural ways of knowing, instruction, and learning, which has fostered the popularity of culturally relevant, responsive, and sustaining pedagogies^57–59^. These pedagogies are linked in the ways they foreground students’ cultural experiences in the classroom and how they work to develop a critical (sociopolitical) consciousness^47^, whereby classroom instruction provides opportunities for students to critique the injustices within their social world.

Recently, intervention studies have shown the impact of cultural and critical pedagogies on Black American adolescent outcomes. Nevertheless, these studies are scarce and tend to demonstrate effects for either racial-ethnic identity development^60,61^ or achievement^62^, with none considering how students negotiate both simultaneously. Furthermore, these studies tend to be at the classroom level, rather than demonstrating effectiveness broadly across different types of schools.

In sum, both institutional and interpersonal solutions to racial inequality have been proposed and, to some extent, studied for their effectiveness. Magnet schools have been examined for their effectiveness at reducing racial isolation and promoting positive intergroup relations^44,45^, but these studies have revealed mixed results regarding academic performance and none has examined psychological and academic outcomes together. Similarly, studies on the effectiveness of cultural and critical pedagogies have addressed racial-ethnic identity and academic performance separately but not in tandem. The current study begins to address these issues by examining how school racial climate as well as cultural and critical pedagogies predict the ways Black American youth negotiate their racial and academic selves in concert, and with a particular focus on resistance motivation as a potentially adaptive coping response to racialized stress in school environments. In the current study, our research questions are:

1. For Black American adolescents, what profiles emerge in how they negotiate their racial identity (i.e., centrality, public regard, and resistance motivation) along with their mathematics identity (i.e., attainment value of mathematics, mastery experiences in mathematics)? Are these negotiation profiles robust over time?
2. Are students’ racial-mathematical negotiation profiles associated with other variables such as their perceptions of school climate (school racial stereotyping and cultural and critical mathematics instruction) and their emotions around mathematics?
3. Does school type (i.e., magnet vs. neighborhood) differentially predict the profiles for Black American adolescents?

## Results

**Fig. 1:**
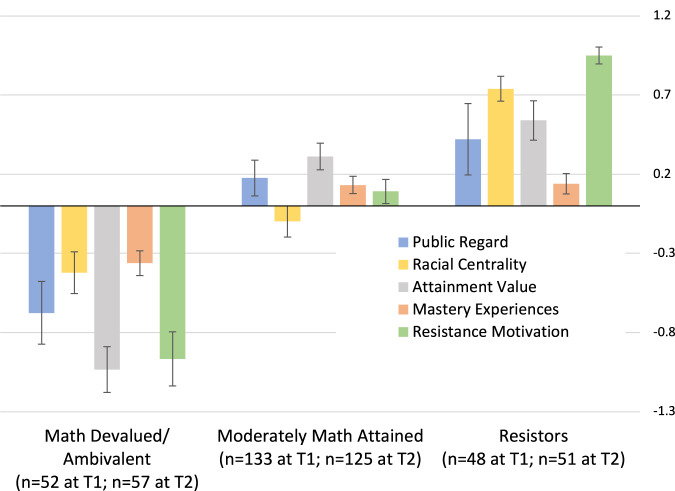
Longitudinal LPA solution with standard error bars.

Raw scores and correlations for the profile indicators are shown in Tables 1 and 2. In reference to our first research question regarding the nature and type of negotiation profiles among Black American adolescents, a three-profile solution had optimal fit across T1 and T2 (illustrated in Fig. 1 and Tables 3 and 4).

**Table 1:** Profile indicators (raw scores)

Measure	Time	Cronbach’s *α*	Scale	Mean	SD
Attainment value	T1	0.79	1–6	4.11	1.19
	T2	0.79	1–6	3.63	1.18
Mastery experiences	T1	0.80	1–6	4.07	1.12
	T2	0.43	1–6	3.69	0.85
Racial centrality	T1	0.77	1–7	4.97	1.28
	T2	0.77	1–7	5.08	1.26
Racial public regard	T1	0.84	1–7	4.21	1.58
	T2	0.84	1–7	3.74	1.48
Resistance motivation	T1	0.90	1–6	4.62	1.25
	T2	0.91	1–6	4.67	1.27

**Table 2:** Auxiliary measures (raw scores)

Measure	Time	Cronbach’s *α*	Scale	Mean	SD
Stereotyping	T1	0.81	1–5	2.43	1.03
	T2	0.80	1–5	2.62	0.99
Cultural competence	T1	0.88	1–5	2.26	1.13
	T2	0.86	1–5	2.32	1.10
Cultural socialization	T1	0.84	1–5	1.83	1.07
	T2	0.91	1–5	1.99	1.20
Critical consciousness	T1	0.85	1–5	2.15	1.10
	T2	0.86	1–5	2.16	1.09
Negative math emotions	T1	0.85	1–6	2.84	1.12
	T2	0.72	1–6	2.98	1.05

**Table 3:** Correlations at T1 (*n* = 177)

		(1)	(2)	(3)	(4)	(5)	(6)	(7)	(8)	(9)	(10)	(11)
(1)	CCMI	1.00										
(2)	Negative math emotions	0.08	1.00									
(3)	Stereotyping	0.00	0.16*	1.00								
(4)	Public regard	0.26***	0.07	−0.04	1.00							
(5)	Racial centrality	0.12	0.13	0.13	0.31***	1.00						
(6)	Attainment value	0.28***	−0.18*	0.03	0.30***	0.27***	1.00					
(7)	Mastery experiences	0.17*	−0.46***	−0.11	0.05	0.01	0.41***	1.00				
(8)	Resistance motivation	0.15*	0.00	−0.02	0.20**	0.26***	0.43***	0.24**	1.00			
(9)	Age (months)	−0.07	0.12	0.14	−0.12	−0.15*	−0.11	0.09	−0.14	1.00		
(10)	Sex (female = 1)	−0.02	0.10	0.04	−0.07	0.04	−0.05	0.07	0.14	0.05	1.00	
(11)	Magnet (magnet = 1)	−0.28***	−0.13	0.20**	−0.43***	−0.24**	−0.32***	0.04	−0.18*	0.23**	0.06	1.00

*p < 0.05; **p < 0.01; ***p < 0.001.

**Table 4:** Correlations at T2 (*n* = 190)

		(1)	(2)	(3)	(4)	(5)	(6)	(7)	(8)	(9)	(10)	(11)
(1)	CCMI	1.00										
(2)	Negative math emotions	−0.01	1.00									
(3)	Stereotyping	−0.08	0.13	1.00								
(4)	Public regard	0.28***	−0.09	−0.25***	1.00							
(5)	Racial centrality	−0.03	−0.01	−0.01	0.09	1.00						
(6)	Attainment value	0.31***	−0.22**	−0.15*	0.32***	0.23**	1.00					
(7)	Mastery experiences	0.16*	−0.10	−0.02	0.15*	0.20**	0.34***	1.00				
(8)	Resistance motivation	0.06	−0.09	−0.14	0.21**	0.30***	0.38***	0.18*	1.00			
(9)	Age (months)	−0.07	0.14	0.12	−0.11	−0.16*	−0.07	0.10	−0.08	1.00		
(10)	Sex (female = 1)	−0.23**	0.15*	0.06	−0.11	0.14	−0.08	−0.07	0.13	0.01	1.00	
(11)	Magnet (magnet = 1)	−0.29***	0.05	0.37***	−0.30***	−0.14*	−0.22**	−0.06	−0.17*	0.21**	0.11	1.00

*p < 0.05; **p < 0.01; ***p < 0.001.

Profile 1, which we labeled *Mathematics Devalued/Ambivalent*, was characterized by relatively low mathematics attainment value and resistance motivation (both one SD below the mean). The mean response for mathematics attainment value was in the “disagree” portion of the scale at both T1 and T2 (see Table 5 for all within-profile predicted raw scores). Racial centrality was 0.45 SD below the overall sample mean but the mean predicted raw score was still above the scale midpoint, corresponding with an “agree” response. The mean resistance motivation score corresponded with a “neutral” response; we interpret this as ambivalence toward the idea of resisting racial stereotypes by excelling in mathematics. Finally, racial public regard was well below the scale midpoint in this profile, a result which we discuss further below.

**Table 5:** Profile indicators: predicted raw scores for longitudinal model

Measure	Time	Devalued/Ambivalent [95% CI]	Diff^a^	Moderately attained [95% CI]	Diff^a^	Resistors [95% CI]	Diff^a^	Scale
Attainment value	T1	3.05 [2.77,3.34]	−0.45	4.42 [4.25,4.59]	0.92	4.65 [4.40,4.90]	1.15	1–6
	T2	2.57 [2.28,2.86]	−0.93	3.94 [3.78,4.11]	0.44	4.17 [3.92,4.42]	0.67	1–6
Mastery experiences	T1	3.74 [3.59,3.88]	0.24	4.19 [4.10,4.29]	0.69	4.20 [4.08,4.32]	0.70	1–6
	T2	3.41 [3.29,3.53]	−0.09	3.79 [3.71,3.88]	0.29	3.80 [3.70,3.90]	0.30	1–6
Racial centrality	T1	4.54 [4.30,4.79]	0.54	4.85 [4.67,5.03]	0.85	5.64 [5.49,5.78]	1.64	1–7
	T2	4.60 [4.31,4.89]	0.60	4.97 [4.75,5.18]	0.97	5.91 [5.74,6.08]	1.91	1–7
Racial public regard	T1	3.61 [3.26,3.96]	−0.39	4.37 [4.17,4.57]	0.37	4.59 [4.20,4.98]	0.59	1–7
	T2	3.09 [2.70,3.47]	−0.91	3.92 [3.71,4.14]	−0.08	4.16 [3.73,4.60]	0.16	1–7
Resistance motivation	T1	3.59 [3.24,3.94]	0.09	4.70 [4.54,4.86]	1.20	5.60 [5.49,5.71]	2.10	1–6
	T2	3.55 [3.16,3.93]	0.06	4.77 [4.59,4.94]	1.28	5.76 [5.65,5.88]	2.27	1–6

^a^Difference of mean score and scale midpoint. Profiles at T1 and T2 have equal mean factor scores; predicted raw scores and predicted 95% CIs use path coefficients for T1 and T2 measurement models respectively.

Profile 2, which we labeled *Moderately Math Attained*, was characterized by attainment value 0.3 SD above the mean and mastery experiences 0.2 SD above the mean, with other indicators not significantly different from the mean. In terms of scale score, nearly all the indicators were moderately above the midpoints of their respective scales (e.g., “Agree a little”), reflecting a slight positive orientation to each.

Profile 3, labeled *Resistors*, was characterized by resistance motivation one SD above the mean, along with high racial centrality and mathematics attainment value (0.8 and 0.6 SD above the mean respectively). Their above-average sense of racial centrality, math attainment, and resistance motivation truly characterizes the ethos of psychological resistance to oppression and pejorative narratives (i.e., stereotypes) about their racial-ethnic group. Their Public Regard measure was higher, on average, than in Profile 1, but relatively close to the “neutral” point on the scale (see Table 5). The high dispersion of this measure may indicate a variety of cognitive associations evoked by the questionnaire prompts.

### Profile predictors

Using the R3STEP procedure in Mplus (a multinomial logistic regression of auxiliary variables on profile membership probabilities) we tested several covariates for association with profile membership: reported levels of cultural and critical mathematics instruction (CCMI) and school racial stereotyping, reported levels of negative emotions around mathematics, and age and biological sex. These auxiliary variables, measured at each time point, were tested against the profiles measured at the same times (see the “Methods” section for further detail). Raw scores for the continuous auxiliary variables are shown in Table 2. Correlations for all variables are shown in Tables 3 and 4. Results of the multinomial logistic regression are shown in Tables 6 and 8.

**Table 6:** Multinomial logistic regression of covariates on profile membership at T1

	Coef.	SE	Odds ratio
Resistors (*n* = 48)			
Cultural and critical mathematics instruction	0.597	0.467	1.817
Stereotyping at school	0.506	0.504	1.659
Magnet school status (1 = magnet; 0 = non-magnet)	−1.808*	0.923	0.164
Negative math emotions	−0.677^†^	0.398	0.508
Age (months)	−0.031^†^	0.019	0.970
Sex (1 = female; 0 = male)	0.641	0.660	1.898
Moderately attained (*n* = 99)			
Cultural and critical mathematics instruction	0.717^†^	0.380	2.047
Stereotyping at school	0.506	0.377	1.745
Magnet school status (1 = magnet; 0 = non-magnet)	−2.216**	0.774	0.109
Negative math emotions	−0.661*	0.277	0.516
Age (months)	−0.008	0.014	0.992
Sex (1 = female; 0 = male)	1.162*	0.575	3.197

Reference class is P1 (Devalued/Ambivalent, *n* = 52). Odds ratios for continuous covariates are based on covariate factor scores. Profile counts represent most likely profile membership.

^†^*p* ≤ 0.1; *p ≤ 0.05; **p ≤ 0.01.

#### Background variables

Sex was significantly associated with profile membership; age was not. Overall, being female increased the odds of being in a more positively oriented profile. At T1, being female tripled the odds of being in the Moderately Attained profile compared to the Devalued/Ambivalent profile; at T2, being female tripled the odds of being in the Resistor profile compared to the Devalued/Ambivalent profile.

#### Pedagogy

Our second research question posed whether cultural and critical mathematics instruction (CCMI) or school racial stereotyping differently predicted the profiles. The data produced from our measure of cultural and critical mathematics instruction showed an abnormal distribution. In the distribution of raw data, the modal responses were at the bottom of all three subscales; the median response was 1.67 for the subscales averaged together (1 = “not true at all”; 2 = “somewhat not true”), indicating that, by and large, Black students did not see cultural and critical mathematics instruction happening at the schools in this study.

Measurement models for all three subscales had acceptable fit indices (see Table 7). Given the well-established importance of pedagogy for racial and academic identity formation^53,60–62^, we proceeded with using cultural and critical mathematics instruction (CCMI) in our inferential analyses. At T1, a multinomial logistic regression showed a marginally significant association (0.05 < *p* < 0.10) between CCMI and membership in the Moderately Attained profile: increasing CCMI by one standard deviation doubled the odds of being in that profile compared to the Devalued/ Ambivalent profile. At T2, CCMI reached full significance (*p* < 0.05) as a predictor of the Moderately Attained profile, with an odds ratio of over 2.5. At both time points, CCMI was associated with higher odds of being in the Resistor profile compared to the Devalued/Ambivalent profile, but this association did not reach statistical significance (possibly due to sample size).

**Table 7:** Model fit indices: profile indicators, school climate measures, and negative math emotions

Construct	Degrees of freedom		${\chi }^{2}$ *p* value	RMSEA	CFI	TLI	SRMR
Attainment value	T1	2	0.2452	0.045	0.997	0.990	0.017
	T2	2	0.2804	0.036	0.998	0.994	0.018
Mastery experiences	T1	1	0.1862	0.062	0.997	0.982	0.013
	T2	1	0.8775	0.000	1.000	1.000	0.002
Racial centrality	T1	4	0.8128	0.000	1.000	1.000	0.011
	T2	4	0.2094	0.047	0.993	0.982	0.020
Racial public regard^a^	T1	0	n/a	n/a	n/a	n/a	n/a
	T2	0	n/a	n/a	n/a	n/a	n/a
Resistance motivation	T1	6	0.1051	0.064	0.992	0.981	0.018
	T2	6	0.2651	0.037	0.998	0.995	0.016
Stereotyping	T1	3	0.3840	0.000	1.000	1.000	0.010
	T2	3	0.3550	0.013	1.000	0.999	0.012
Cultural competence	T1	4	0.6625	0.000	1.000	1.000	0.010
	T2	4	0.7878	0.000	1.000	1.000	0.009
Cultural socialization^a^	T1	0	n/a	n/a	n/a	n/a	n/a
	T2	0	n/a	n/a	n/a	n/a	n/a
Critical consciousness	T1	1	0.6739	0.000	1.000	1.000	0.003
	T2	1	0.0338	0.129	0.990	0.943	0.016
Negative math emotions	T1	7	0.0514	0.072	0.985	0.967	0.031
	T2	7	0.6626	0.000	1.000	1.000	0.020

^a^A three-indicator construct (just identified).

#### Stereotyping

We did not find student perceptions of racial stereotyping in school to be directly associated with profile membership. This does not rule out possible indirect effects of stereotyping that were not measured in this study; see the Discussion for suggested directions for further research on this topic.

#### Math-related emotions

We hypothesized that math-related emotions would be associated with profile membership. A multinomial logistic regression showed significant associations for the Moderately Attained profile at both time points and for the Resistor profile at T2 (also marginal significance at T1, with $0.05 < p < 0.10$) At T2, students who were one standard deviation above the mean on negative math emotions were less than half as likely to be in the Moderately Attained or the Resistor profile, compared to their odds of being in the Devalued/Ambivalent profile.

### Magnet school environments

**Fig. 2:**
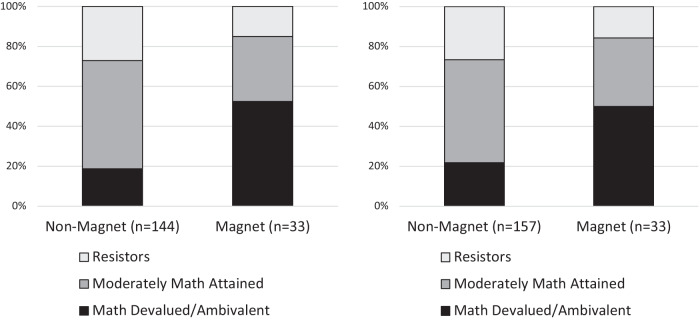
School profile composition by school type at T1 (left) and T2 (right).

Our final research question sought to understand whether school type (i.e., magnet vs. neighborhood schools) differentially predicted the profiles. We found profile membership to be significantly associated with school type (see Tables 6 and 8). We exported most likely class membership from the longitudinal model (see Table 9) and conducted a Pearson chi-square test for categorical association with school type (${\chi }_{{\rm{T1}}}^{2}=19.06,p < 0.001;{\chi }_{{\rm{T2}}}^{2}=12.45,p < 0.01$), illustrated in Fig. 2. A post hoc multiple comparisons test revealed that the Devalued/Ambivalent profile was significantly overrepresented in magnet schools compared to neighborhood schools (significance at the 0.01 level with the Bonferroni correction).

**Table 8:** Multinomial logistic regression of covariates on profile membership at T2

	Coef.	SE	Odds ratio
Resistors (*n* = 51)			
Cultural and critical mathematics instruction	0.537	0.379	1.710
Stereotyping at school	−0.155	0.322	0.856
Magnet school status (1 = magnet; 0 = non-magnet)	−1.631*	0.726	0.196
Negative math emotions	−0.825*	0.335	0.438
Age (months)	0.021	0.015	1.022
Sex (1 = female; 0 = male)	1.132*	0.577	3.102
Moderately attained (*n* = 102)			
Cultural and critical mathematics instruction	0.926**	0.345	2.524
Stereotyping at school	−0.035	0.336	0.966
Magnet school status (1 = magnet; 0 = non-magnet)	−1.553^†^	0.822	0.212
Negative math emotions	−0.794*	0.366	0.452
Age (months)	0.026^†^	0.014	1.026
Sex (1 = female; 0 = male)	0.396	0.509	1.485

Reference class is P1 (Devalued/Ambivalent, *n* = 52). Odds ratios for continuous covariates are based on covariate factor scores. Profile counts represent most likely profile membership.

^†^*p* ≤ 0.1; *p ≤ 0.05; **p ≤ 0.01.

**Table 9:** Most likely profile membership by magnet school status, T1 and T2

	Time 1		Time 2	
	Non-magnet	Magnet	Non-magnet	Magnet
Math Devalued/Ambivalent (P1)	29 (19%)	21 (53%)	36 (22%)	19 (50%)
Moderately Math Attained (P2)	84 (54%)	13 (33%)	85 (52%)	13 (34%)
Resistors (P3)	42 (27%)	6 (15%)	44 (27%)	6 (16%)
Totals	155 (100%)	40 (100%)	165 (100%)	38 (100%)

Counts in Table 6 include imputed most likely profile membership from the longitudinal model.

Due to this association, we sought to understand how the profile covariates might vary over school type. We conducted a multivariate analysis of variance (MANOVA) of all continuous covariates on school type, first verifying that sex was not significantly associated with school type (Pearson ${\chi }^{2}=1.26,p > 0.05$ at T1; Pearson ${\chi }^{2}=2.62,p > 0.05$ at T2). The MANOVA showed significant differences across groups, both at T1 (Lawley–Hotelling trace = 0.47, *F*(4,75) = 8.77, $p < 0.05$) and at T2 (Lawley–Hotelling trace = 0.75, *F*(4,71) = 13.26, $p < 0.05$). We then conducted a step-down analysis to identify the variables for which there were differences.

#### Cultural and critical pedagogy

Reported cultural and critical mathematics instruction (CCMI) was significantly lower in magnet schools at both time points (see Tables 10 and 11). The R3STEP procedure in Mplus does not allow for direct testing of mediation with auxiliary variables; however, the associations of CCMI with profile membership and CCMI with school type point to CCMI as one possible mediator of the relationship between school type and profile distribution.

**Table 10:** Step down analysis of T1 covariates and magnet school status (*n* = 40 per group)

Dependent variable	df	df error	*F*	Eta2	Adjusted means	
Age (years)	1	78	7.89*	0.09	Magnet	16.54
					Non-magnet	15.74
Negative math emotions	1	77	0.01	0.00	Magnet	−0.100
					Non-magnet	−0.077
Cultural and critical math instruction	1	76	17.81*	0.19	Magnet	−0.448
					Non-magnet	0.177
Stereotyping at school	1	75	5.97^†^	0.07	Magnet	0.383
					Non-magnet	−0.103

^†^*p* < 0.025, approaching significance; *p < 0.013, significant at Bonferroni-corrected 0.05 alpha level. No covariate significantly adjusted the mean for other covariates.

**Table 11:** Step down analysis of T2 covariates and magnet school status (*n* = 38 per group)

Dependent variable	df	df error	*F*	Eta2	Adjusted means	
Age (years)	1	74	4.01	0.05	Magnet	17.56
					Non-magnet	16.93
Negative math emotions	1	73	0.03	0.00	Magnet	0.088
					Non-magnet	0.054
Cultural and critical math instruction	1	72	32.66*	0.31	Magnet	−0.608
					Non-magnet	0.409
Stereotyping at school	1	71	9.93*	0.11	Magnet	0.740
					Non-magnet	0.012

*p < 0.013, significant at Bonferroni-corrected 0.05 alpha level. No covariate significantly adjusted the mean for other covariates.

#### Stereotyping

Reported racial stereotyping was higher in magnet schools at both time points, approaching significance at T1 and reaching statistical significance at T2 (see Table 11). Although we did not find an association between stereotyping and profile membership, we note the difference in stereotyping by school environment because it is important data about the magnet school environments represented in this sample. Along with this finding, we note that Public Regard was negatively correlated with magnet school status (see Tables 3 and 4). This underlines the idea that students in magnet schools may have been brought face to face with the reality of racial stereotyping through their experiences in school.

#### Math-related emotions

At both time points, negative math emotions were not significantly different, on average, for magnet school students and non-magnet school students. Given that the magnet schools represented in this sample were STEM-themed schools aimed to attract students interested in mathematics and science, one might expect negative math emotions to be less prevalent in magnet schools. However, the association between negative math emotions and profile membership, together with the overrepresentation of the Devalued/Ambivalent profile in these magnet schools, may help to explain the similar levels of negative math emotions in magnet and non-magnet schools.

## Discussion

This study investigated the racial-mathematical identity profiles of Black American adolescents. Our data revealed three distinct identity profiles: (1) Math Devalued/Ambivalent, (2) Moderately Math Attained, and (3) Resistors. These profiles were robust across time and their contours were stable. Moreover, profile membership was associated with school type, cultural and critical mathematics instruction (T2 only), and math-related emotions. Of the five profile indicators we used, Resistance Motivation was the only one combining ideas about race and mathematics in a single scale; the remaining four indicators treat these topics separately but help to paint a fuller picture of the characteristics of each profile.

The Math Devalued/Ambivalent profile was characterized by low mastery experiences for self-efficacy (a proxy for expectancy) and low mathematics attainment value, making it unsurprising based on EVT that these students did not express a motivation to resist negative stereotypes about Black people by excelling in mathematics. The fact that racial centrality was lowest in this profile (although still above the raw scale midpoint) points away from the “acting white”^34^ premise that some students eschew school success for the sake of maintaining Black identity. Rather, we surmise issues such as low teacher support^63^, performance goal-oriented classrooms^64^, and/or a lack of meaningful, non-rote mathematics instruction^1^ may explain the development of this profile, although we did not test for these directly. The Moderately Math Attained profile held higher mathematics mastery experiences and attainment value, which likely reinforced their achievement motivation. These students valued their Black racial identity more highly than the Devalued/ Ambivalent group: their racial centrality, which was near the sample mean, translated to a raw score of about one point above the scale midpoint (see Table 12).

**Table 12:** Factor score means and variances for longitudinal latent profile indicators

Profile	Indicator	Mean		Difference from mean/pooled SD^a^	Variance	
					T1	T2
Math Devalued/Ambivalent	Public regard		−0.676	−0.485	2.018	1.505
	Racial centrality		−0.422	−0.449	1.516	1.032
	Attainment value		−1.034	−1.067	0.457	0.634
	Mastery experiences		−0.362	−0.500	0.755	0.172
	Resistance motivation		−0.967	−0.952	1.571	1.311
Moderately Math Attained	Public regard		0.176	0.126	2.018	1.505
	Racial centrality		−0.098	−0.104	0.777	0.521
	Attainment value		0.313	0.323	0.457	0.634
	Mastery experiences		0.132	0.182	0.755	0.172
	Resistance motivation		0.091	0.090	0.419	0.308
Resistors	Public regard		0.421	0.302	2.018	1.505
	Racial centrality		0.740	0.787	0.189	0.103
	Attainment value		0.540	0.557	0.457	0.634
	Mastery experiences		0.140	0.193	0.755	0.172
	Resistance motivation		0.950	0.935	0.161	0.012

^a^Using pooled standard deviation for full sample at T1 and T2.

The Resistor profile was characterized by high valuing of mathematics and high resistance motivation, as described by O’Connor^40,65^ and Perez^9^. Despite the psychological literature’s many examinations of negative coping responses to racialized stress among Black Americans (e.g., academic devaluation, racial devaluation) this study is among few to quantitatively validate the existence of adaptive and liberatory psychological coping responses. These students had the strongest sense of Black identity, as evidenced by their expression of racial centrality. Students in this profile had no more experiences of mastery than students in the Moderately Math Attained profile, but, like Shayla, quoted in the introduction, they maintained exceptionally high motivation to succeed. Contrary to our expectations, however, their mean public regard was the highest across the three profiles. This was surprising because of our initial assumption that critical consciousness in adolescents would go hand in hand with a clear-eyed recognition of societal prejudice against the Black community^66^. We interpret this result by noting, first, that in magnet schools Resistors were underrepresented and Devalued/Ambivalent students were overrepresented and, second, that Public Regard was negatively associated with being in a magnet school (see Tables 3 and 4). Taking these results together with the fact that CCMI was lowest in magnet schools (Table 10), we hypothesize that racially diverse school environments like these may accelerate an awareness of racial stereotypes without supplying the resources to critically engage with them and develop resistance motivation.

Public Regard was also widely dispersed in this profile, (see Tables 3 and 4), suggesting that some of these students were emerging into consciousness of societal racial stereotypes, while others may have been unaware of these stereotypes up to this point in their lives. Resistance Motivation may reflect differing nuances of the desire to resist prejudice by excelling in mathematics—perhaps parallel to different types of stereotype threat^67^. A desire to resist threats of racial prejudice against oneself might align with low Public Regard (“I don’t want to be judged negatively based on society’s low opinion of Black people”), whereas a desire to be a positive representative for Black people could be consistent with a moderate or high public regard (“My community has earned respect through decades of struggle; I want to do my part”).

The contours of this profile may suggest that high racial centrality and resistance motivation can develop before, after, or alongside a clearly articulated consciousness of societal racial prejudice. This underscores the need for adults’ emotional sensitivity for engaging the work of consciousness-raising among Black children^68^. Theories of culturally relevant and emancipatory education emphasize warmth and positivity, naming Blackness as good in parallel with their advocations for a critical race analysis of social inequities^58,69–71^. Moreover, it may be that learning about racial prejudice can affect children differently depending on how they encounter this information (i.e., learning from a trusted, same-race adult vs. learning on their own, through traumatic personal experiences).

Our second research question addressed what variables were associated with profile membership. First, we found that CCMI predicted profile membership: combining cultural competence (situating mathematics in a cultural frame), cultural socialization (learning about one’s own culture in a mathematics class), and critical consciousness (learning about the dynamics of structural racism in a mathematics class) predicted membership in the Moderately Attained and Resistor profiles. We interpret this result with caution given that it reached statistical significance only at T2. An adapted measure of CCMI that is calibrated to its very low overall level in high school mathematics may be desirable for future studies.

The absence of an association between profiles and self-reported stereotyping at school may be interpreted in a number of different ways; the present study cannot speak definitively to these possible interpretations. It may be that having an awareness of stereotyping at school acts as a buffer to the psychological effects of being stereotyped. Kendrick, a low-tracked student in McCardle’s magnet school study^20^, took the blame on himself for being “mostly lazy in that I don’t have the determination some people have. I try to do better, but I don’t have that much” (p. 298). Jeremy, on the other hand, said that if being excluded from the IB program (the school’s elite academic track) had any advantages, it would be “the advantage of surprise” (p. 300), i.e., surprising the people who thought he could not do as well as the (mostly White) students in the IB program. Although these two boys were in the same school, Jeremy’s awareness of being stereotyped at school had led him to adopt an attitude of resistance.

However, if it is an indicator of racialized academic tracking in a school, student-reported stereotyping might also correlate with a lack of access to high-quality, rigorous mathematics instruction. This would have to be determined using data that most schools typically do not publish: the representation of Black students in their more prestigious, elite mathematics classes. Resistance motivation without this kind of access would likely be insufficient to increase mathematics performance. We believe further research is necessary to determine how Black students’ experiences of racial stereotyping at school interact with their resistance motivation, experience of mathematics, and academic performance.

We also found that students’ self-reported gender was associated with profile membership. Compared to the likelihood of being in the Devalued/Ambivalent profile, girls were more likely than boys to be Moderately Attained (significant at T1), or Resistors (significant at T2). Contrary to popular thought, this finding aligns with previous research that illustrates that girls tend to outperform boys in secondary mathematics grades and classroom activities, while boys only slightly outperform girls in standardized measures of mathematics^72,73^. However, most of that work has not included girls of color within their samples. Thus, this finding also warrants further research. It may be that adolescent girls of color are already well practiced at coping with prejudice in mathematics classrooms because they have faced gender as well as racial stereotypes in mathematics in the past. One thing is certain: African-American women, who were among the first to educate ex-slaves^74,75^ and were deeply involved in the struggle for school desegregation^19^, may have much to teach us about resistance and fighting for educational opportunity.

Finally, the association between profile and school type provides quantitative validation to the extant historical and qualitative critiques of urban magnet school environments^20,42,44,46,47^. Though masked by the rhetoric of diversity, racialized cost experiences^11,12^ within urban magnet schools remain. Moderately Attained students and Resistors were underrepresented in the magnet schools in this study—environments where Black students were outnumbered, and cultural and critical mathematics instruction (CCMI) was rarest. Given that racial stress can inhibit the use of ordinary stress coping mechanisms^76^, learning mathematics may be especially discouraging for Black adolescents who are racially minoritized in their schools. Moreover, the higher level of stereotyping in the magnet schools in this study should be an additional matter of concern to educators, policymakers, and families, and warrants further study.

Meanwhile, in schools like the predominantly Black neighborhood schools where there was more evidence of positive racial-academic response patterns emerging, educational and college-directed resources remain notoriously scarce^18,77^. In spite of this, the cadre of Resistors we identified shows us that resilience and determination are still possible and that, with proper support, Black adolescents can both embrace their communities and simultaneously prize success in mathematics^40,78^.

Academically selective magnet schools tout the benefits of student body diversity and increased opportunity for Black students. But with racial diversity comes the potential for racialized cost experiences in mathematics: being overlooked for high-track mathematics classes, being the lone Black student in those classes, or facing a school atmosphere of racial stereotyping. This underscores the need for additional racial-mathematical identity supports, especially in school environments that resemble the racially diverse magnet schools represented in this study. A partial solution may lie in the crafting of intentional spaces where Black adolescents “learn and practice mathematics, develop strong mathematics identities themselves, and are inducted into a community of mathematics doers”^79^. For example, the Benjamin Banneker Association provides resources for school-sponsored mathematics clubs geared toward Black youth^80^.

Based on our findings, CCMI in the mathematics classroom may also play a role in increasing Black students’ resistance motivation. However, our questionnaire prompts on this measure met with general disagreement across both school types, indicating that there were few, if any, exemplars of this kind of instruction across the schools in our sample. Given the existence of well-developed *general* curricular frameworks for cultural and critical instruction^81–83^, this raises the important question of why it appears to be so scarce in high school *mathematics* classrooms. Research addressing this question could benefit the teachers, curriculum designers, and educational leaders who see the benefits of CCMI and would like to foster its use at this important stage in students’ lives.

The chief limitations of this study stemmed from the difficulty of obtaining these data from a large sample of Black adolescents. A larger Black student sample might have produced more robust results (some of our results were reported at the 10% significance level) and might also have allowed us to test profile covariates across time. For such an analysis we would have needed complete data at both time points. Our already small within-profile sample size did not allow leeway for missing data, but unfortunately we did have missing data from students who left or joined the study between time points. In particular, we would have liked to include grades and standardized test scores in our analysis, but we were not able to collect those data for some participants, and therefore had the most missing data on those variables. We consider this study to be a first foray into a set of urgent questions that are not easy to investigate. Additionally, generalizability is likely limited given the focus on one school district. However, the existing research on segregated schools and magnet schools is geographically widespread and suggests that the results our data yielded may not be unique. More work is needed in other districts, both in the U.S. and internationally.

## Methods

### Participants

The data for this study spanned two consecutive school years (2017–2018, and 2018–2019), which were the final 2 years (waves four and five) of a 5-year study across five secondary schools in one large northeast U.S. city^1^. According to the U.S. Census Bureau^84^, 49.5% of the city’s residents identified as Black or African American, the median income was $37,476 and 26.3% of the population lived at or below the poverty line. Schools in this city’s metropolitan area retained the second highest level of Black-White school segregation in the nation^85^.

Students were recruited through in-person announcements in mathematics classrooms across all five schools. Three were high schools (grades 7–12 or 9–12) and two were middle schools (grades K-8). Two of the high schools were public magnet schools that ranked within the top 20% of high schools within the state. They maintained 98% and 95% graduation rates and were ~33% and 22% Black American, respectively^86^. The third high school was a “neighborhood” school ranked within the bottom 33% of high schools within the state, maintained a 61% graduation rate, and was ~91% Black American^86^. The two middle schools were also neighborhood schools, over 90% Black American, and were generally low-performing schools feeding into the predominantly Black high schools in the city^87^.

The response rate for student assent and guardian consent was ~64%, which resulted in a sample of 285 students who self-identified as Black or African American at the beginning of the study in 2014 (*M* age = 12.75 years; 50.6% female). Of the students who left the schools we were able to follow some to their new schools, but the unavoidable turnover in the sample resulted in 197 and 210 participants in waves four and five respectively (T1 and T2 in this study). The total number of participants in the present analyses was 225: 41 in magnet schools and 184 in non-magnet schools.

### Data collection

Racial and mathematics beliefs were measured via survey questionnaires administered in the spring of the 2017–2018 school year (T1) and the spring of the 2018–2019 school year (T2). Math-related emotions were measured at each time point, along with perceptions of school climate (i.e., stereotyping) and classroom pedagogy (cultural and critical mathematics instruction). Student questionnaires were completed online in the school computer labs using individual computers. Typically, 10 to 25 students were surveyed at a time, and on average it took 34 min for students to complete the battery of survey questions. Survey questions were not randomized in their presentation. Trained undergraduate and masters research assistants under the direction of the PI and second author (a Black American man) monitored the survey administration and answered students’ questions as needed but did not interact with students in any additional ways as they completed the survey items. The research team shifted in size, from 5-8 members, over the years of this study and was racially-ethnically diverse with Black, Latane, and White American research assistants.

#### Student and classroom questionnaires

##### Profile indicators

We chose five indicator constructs to identify students’ beliefs and motivational response patterns to racial and mathematics identification: attainment value for mathematics, mastery experiences of self-efficacy in mathematics, racial centrality, racial public regard, and resistance motivation. Raw scores for these indicators are shown in Table 1. For our final analysis we used factor scores (see Table 7 for model fit indices). Summed or averaged raw scores would have disregarded the strength of item loading on each factor and the different metrics of the observed variables; factor scores (based on a tested measurement model) account for these^88^. Correlations for the five profile indicators across our full sample at T1 and T2 are shown in Table 2.

*Attainment value* of mathematics refers to the importance students attach to the domain of mathematics as they view it as self-defining or a reflection of their identity. It was measured through the mean of four questionnaire items (e.g., “being a good math student is an important reflection of who I am”). Student participants evaluated these items for themselves on a six-point Likert scale, from 1 (Completely Disagree) to 6 (Completely Agree), with higher scores reflecting higher attainment value.

*Mastery experiences* in mathematics are a source of self-efficacy (in EVT terms, high expectancy for success) for many students, as experiences of mastery within mathematics support students’ belief in their mathematical abilities^7^. Six items adopted from Usher and Pajares^7^ measured students’ mastery experiences (e.g., “I got good grades in math on my last report card”) using a Likert-type response scale ranging from 1 (definitely false) to 6 (definitely true). Reliability and validity for this measure have been established across varied samples in previous research^7^; however, the scale did not show the expected internal consistency for our sample at T2 (Cronbach’s $\alpha =0.43$). Nevertheless, we fit the same measurement model for the construct at both waves with acceptable fit and without reducing it to a just-identified model (see Table 7).

*Racial centrality* refers to the importance an individual ascribes to their race as a self- defining feature of their personhood^6^. This indicator was measured using the racial centrality subscale of the Multidimensional Inventory of Black Identity (MIBI^6^), which contains eight Likert-type items (e.g., “Being Black is an important reflection of who I am”). It was scaled from 1 (Strongly Disagree) to 7 (Strongly Agree), where higher scores reflect greater racial centrality. The subscale has shown strong psychometric properties across dozens of studies in middle school, high school^89^, and college^6^.

*Racial public regard* refers to an individual’s perception of how others (i.e., society) view their racial group, whether positively or negatively. Public regard was measured using the public regard subscale of the MIBI^6^, which contains six Likert-type items (e.g., “In general, others respect Black people”). It is scaled from 1 (Strongly Disagree) to 7 (Strongly Agree), where higher scores reflect perceptions that Black Americans are viewed more positively by others. The scale has been well-validated in previous research^6,90^.

*Resistance motivation* reflects an individual’s drive for school success as a way to resist discrimination, stigma, and stereotypes against one’s racial group^9^. Seven items measured resistance motivation, each scaled on a six-point Likert scale (e.g., “I want to do well in math to prove to others that people like me can be successful”; “I want to do well in math to challenge negative images about people of my race”; 1 = Completely disagree, 6 = Completely agree). Since the initial validation of this scale was based on Latinx immigrant students, we conducted a confirmatory factor analysis with the present sample of Black American adolescents (see Table 7 for fit indices).

##### Profile covariates

We measured several covariates at each time point. Our school climate measures and negative math emotions scale were all taken from previously-validated scales. We verified strong internal consistency for each scale using Cronbach’s alpha (Table 2). For our final analysis with these variables we used factor scores (see Table 7 for measurement model fit indices). As background variables, we also collected data on age and students’ self-reported gender.

##### Stereotyping

Byrd^91^ developed and validated the School Climate for Diversity-Secondary scale, which is a grades 5-12 survey instrument examining interracial interactions and racial socialization within a school’s culture. The *Stereotyping* subscale measures the degree to which students perceive that racial and cultural stereotypes (from students, teachers, administrators, or curriculum materials) are endorsed within the school. The subscale contains five Likert-type items (e.g., “students here have a lot of stereotypes about your racial or ethnic group;” “Your racial or cultural group is represented in stereotypical ways in textbooks and class materials”) measured on a 5-point scale (1 = Not at all true; 5 = Completely true). For our final analysis we used factor scores.

##### Critical and cultural mathematics instruction (CCMI)

The *Cultural Competence, Cultural Socialization*, and *Critical Consciousness* subscales, also from Byrd’s^91^ School Climate for Diversity-Secondary scale, measured the degree to which students perceived classroom instruction discussed issues of race and culture (i.e., cultural competence and cultural socialization), and developed their understanding of power and privilege as it pertained to racism and systemic oppression (i.e., critical consciousness). Given the specific context of this research to mathematics instruction, we adapted the scale items to reference students’ mathematics classrooms. Sample items included “You have learned about new cultures and traditions in your math class(es) at your school.” (i.e., cultural competence), “In your math class(es) at your school, you have participated in math activities that teach you more about your cultural background” (i.e., cultural socialization), and “In your math class(es), you have learned about how race/ethnicity plays a role in who is successful” (i.e., critical consciousness). These three scales used a five-point Likert scale (1 = Not at all true; 5 = Completely true).

We combined the three subscales, specifying a construct of cultural and critical mathematics instruction (CCMI) measured by the three latent variables *Cultural Competence, Cultural Socialization*, and *Critical Consciousness*.

##### Math-related emotions

Negative math emotions, conceptualized as anxiety, stress, and fatigue, can also inform self-efficacy in mathematics^7,92^. Students may interpret negative emotional arousal as foreshadowing failure or indicating low ability. Six Likert-type items measured students’ negative emotional state (e.g., “I start to feel stressed-out as soon as I begin my math work;” “My whole body becomes tense when I have to do math”), each scaled from 1 (definitely false) to 6 (definitely true).

#### Ethical research statement

We have complied with all ethical regulations for conducting the present research. We have obtained informed consent from all participating individuals and their families. Montclair State University IRB approved this study in addition to the IRB office/committee of the partner school district.

### Data analysis

#### Latent profile analysis (LPA)

Latent profile analysis (LPA) is an exploratory quantitative technique aimed toward identifying distinct groups/patterns of how a set of continuous variables relate to one another within a given sample. These groups are typically called “profiles,” and LPA produces probability estimates of the likeliness that a certain individual/case belongs to a given profile as well as the optimal number of profiles within a sample. For these analyses, we employed mixture modeling in Mplus (Mplus 8.1)^93^.

##### Measurement models

We used the following five profile indicators for our analysis: mathematics mastery experiences, mathematics attainment value, racial centrality, racial public regard, and resistance motivation. For these five indicators, we first tested the goodness of fit of each measurement model at each time point. We ensured that (1) each measurement model had acceptable fit indices at both time points (see Table 7), and that (2) the measurement models used at T1 and T2 were identical. One example of our model refinement was eliminating reverse-coded items in the measurement scales due to poor item loading and poor overall model fit. Certain other items were also dropped because of poor factor loading, which could be reasonably explained by features of the questionnaire prompts. Allowing the error terms of certain items to correlate (only within scales) also improved model fit. These correlated error terms were reasonably explained by survey design theory–principally, priming effects due to item proximity in the questionnaire^94^. In constructing the LPAs, we used factor scores for all indicator variables.

##### Profile analysis conditions

The default condition for latent profile analyses in Mplus is that the indicator means are freely estimated but the indicator variances are constrained to equality across profiles. According to Johnson^95^, these conditions should not always be assumed; rather, allowing indicator variances to be freely estimated may better reflect a given situation. Johnson recommends comparing the fit of models with and without constrained variances. Although freely estimating *all* the indicator variances proved too computationally complex (resulting in uncertainty regarding local maxima) we did allow free estimation of the variances of resistance motivation and racial centrality, because of the skewness of the data distributions for these two indicators. Doing so resulted in better model fit and a more theoretically useful set of profiles, with enough cases in each profile to compare on predictor and outcome variables.

##### Number of profiles

We chose the optimal number of profiles based on the statistical fit indices, including the Akaike Information Criterion (AIC), Bayesian Information Criterion (BIC), and sample size adjusted Bayesian Information Criterion (aBIC), with smaller values of AIC, BIC, and aBIC indicating better model fit. We also used the Vuong-Lo-Mendell-Rubin likelihood ratio test (VLMR-LRT) to compare each model with k latent classes to a model with k-1 latent classes, with a non-significant *p* value indicating the k-1 model as the better-fitting model^96^. In judging between models with comparable fit indices, we also attended to theoretical interpretability and substantive meaningfulness of the model^97^. We found strong evidence for a 3-profile model separately at both T1 and T2 (see Tables 13 and 14).

**Table 13:** Model fit measurements by number of profiles, T1

Model fit measurements (T1)	2-Profile model	3-Profile model	4-Profile model^a^	5-Profile model^a^
AIC	2754	2708	2681	2674
BIC	2813	2794	2793	2813
aBIC	2756	2712	2685	2680
VLMR-LRT	0.0001	0.2592	0.0054	0.6292
entropy	0.798	0.768	0.834	0.859
Class sizes	58; 141	49; 107; 43	48; 43; 4; 104	48; 4; 3; 41; 103

^a^Non positive-definite first-order derivative product matrix.

**Table 14:** Model fit measurements by number of profiles, T2

Model fit measurements (T2)	2-Profile model	3-Profile model	4-Profile model^b^	5-Profile model^a^
AIC	2509	2449	2428	2410
BIC	2569	2536	2541	2551
aBIC	2512	2454	2434	2418
VLMR-LRT	0.0003	0.069	0.2889	0.9536
entropy	0.656	0.695	0.763	0.818
Class sizes	96; 114	57; 98; 55	53; 55; 100; 2	7; 48; 99; 2; 54

^a^Non positive-definite first-order derivative product matrix.

^b^Non positive-definite first-order derivative product matrix and concern about local maxima (best log-likelihood value not replicated with 10,000 random starting values and 1000 iterations).

##### Longitudinal analysis

**Fig. 3:**
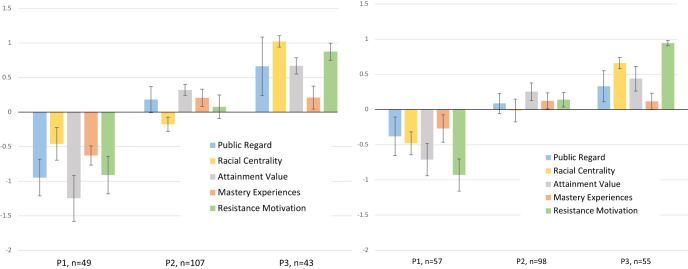
LPA results at T1 (left) and T2 (right) with standard error bars.

LPAs from data collected at two time points may be combined into a single model for longitudinal analysis if similarity between the individual LPAs has been established^98^. In the present analysis we found configural similarity, in that LPAs done at T1 and T2 using the same five indicators each yielded three profiles. Moreover, the profiles showed sufficient similarity in structure (see Fig. 3) to test a constrained model (i.e., constraining the indicator means to equality across time).

The constrained-means model yielded satisfactory model fit: Based on the BIC, the constrained-means model had a better model fit than a combined model with freely estimated means (see Table 15). The entropy measure for the mean-constrained model (0.64) was high enough for us to have reasonable confidence in assigning participants to a single profile at each time point. We did not find dispersion similarity (i.e., better model fit after constraining the within-profile variances to equality) and, therefore, did not extend our analysis to a formal latent profile transition analysis (LPTA). Means and variances for all constructs in the final model are shown in Table 12. To give the reader context for interpreting the profile means, they were converted to adjusted raw scores using the intercepts and path coefficients from the indicator measurement models for T1 and T2 respectively (Table 5).

**Table 15:** Model fit indices by T1–T2 constraints

Combined model fit indices	Unconstrained	Constrained means	Constrained means and variances
AIC	4982	5157	5256
BIC	5368	5284	5353
aBIC	5013	5167	5264

#### Profile covariates

To identify covariates associated with profile membership, we conducted a multinomial logistic regression at each time point using the R3STEP procedure in Mplus, using Profile 1 as the comparison group. The covariates we tested were Cultural and Critical Mathematics Instruction (CCMI), stereotyping at school, magnet school status, negative math emotions, age, and biological sex (see Tables 6 and 8). Each covariate was measured contemporaneously with the profile indicators at both T1 and T2. We tested only the T1 covariates for the T1 profiles and only the T2 covariates for the T2 profiles, because the R3STEP procedure uses listwise deletion; because of turnover in the sample, testing the covariates across time points would have resulted in data loss. For each separate R3STEP procedure, we fixed the profile indicator means and variances to the values obtained from the combined longitudinal model.

#### Post hoc analysis of covariates and school type

Because school type was a significant predictor of profile membership (see Tables 6 and 8), we tabulated most likely profile membership by magnet school status (Table 9) and confirmed the categorical association with a Pearson chi-square test for categorical association (${\chi }_{{\rm{T1}}}^{2}=23.36,p < 0.001;{\chi }_{{\rm{T2}}}^{2}=13.06,p=0.001$).

We were interested in whether the profile covariates (the auxiliary variables listed in Tables 6 and 8) were associated with magnet school status. We conducted a multivariate analysis of variance (MANOVA) of all continuous covariates on school type. MANOVA tests are robust for non-normal distributions if the group sizes are similar^99^. Because our sample had fewer magnet school students than non-magnet school students, we selected a random subsample of non-magnet school students for the MANOVA at each wave. In each subsample we tested for association between magnet school status and sex, and there was no significant overrepresentation of either sex at either time point (Pearson ${\chi }_{{\rm{T1}}}^{2}$ = 1.27, *p* > 0.05; Pearson ${\chi }_{{\rm{T2}}}^{2}$ = 2.62, *p* > 0.05).

The MANOVA showed significant differences across groups, both at T1 (Lawley–Hotelling trace = 0.47, *F*(4,75) = 8.77, $p < 0.05$) and at T2 (Lawley–Hotelling trace = 0.75, *F*(4,71) = 13.26, $p < 0.05$). We then conducted a step-down analysis to identify the variables for which there were differences using the Bonferroni-corrected alpha level of 0.05/4 (for four covariates) to avoid Type 1 error inflation. We first tested for age alone, then for negative math emotions with age as a covariate, then for CCMI with negative math emotions and age as covariates, and finally for stereotyping with CCMI, negative math emotions, and age as covariates. Test statistics, statistical significance, and adjusted factor score means for the step-down analysis are shown in Table 10.

### Reporting summary

Further information on research design is available in the Nature Research Reporting Summary linked to this article.

### Supplementary information

Supplemental Information

Reporting Summary

## Data Availability

The data and survey items used in the current study are available on the Open Science Framework at: https://osf.io/xbvfe/?view_only=767ee696905d4828b47f5181a89cca9d.

## Code availability

The code used in the current study is available on the Open Science Framework at: https://osf.io/xbvfe/?view_only=767ee696905d4828b47f5181a89cca9d.
