# Supplementary material for: Black adolescents’ motivation to resist the false dichotomy between mathematics achievement and racial identity
Source: NPJ Sci Learn. 2024 Mar 2;9:15. doi: 10.1038/s41539-024-00219-9 (PMC10908790; doi:10.1038/s41539-024-00219-9)
Supplement: Supplementary file 1 — Supplemental Information [file 41539_2024_219_MOESM1_ESM.pdf]

Black adolescents' motivation to resist the false dichotomy between mathematics achievement and racial identity

Wilson & Matthews, 2024

Notes & Supplementary Information

i. Pooled standard deviations from T1 and T2 from the entire sample
ii. We dropped one item from the cultural competence subscale because of poor factor loading. For critical consciousness at T2, the χ^2 p value was significant and the RMSEA was slightly high; however, the CFI, TLI, and SRMR all indicated acceptable model fit. We allowed the error terms for some within-factor pairs of items to correlate; in these instances, the items were worded nearly identically or were consecutive in the questionnaire, pointing to possible priming effects.
iii. Of the 210 participants, we had complete grade data for only 156 (74%) and complete standardized test score data for only 84 (40%).
iv. In the U.S. public school system, a student is automatically assigned to a "neighborhood" school (due to urban school closures, these schools are no longer necessarily near the student's home), unless the family chooses to apply to a magnet, charter, or private school.
v. For mastery experiences at T2, there was one non-reverse-coded item eliminated before measuring the Cronbach's alpha of .43. This item mentioned doing well on tests, and we believe the respondents may have thought of standardized test scores rather than in-school tests (the other items on the scale all referred to in-school success). No single item explained the low alpha for the remaining scale. In the measurement model, the path coefficients for the four items we retained were: .486, .312, .477, and .304.
vi. Although Cronbach's alpha was low for this scale at T2, we found a measurement model for this construct using Mplus software; the error terms for two items were correlated. See Table 9 for model fit indices.
