# Supplementary material for: Black adolescents’ motivation to resist the false dichotomy between mathematics achievement and racial identity
Source: NPJ Sci Learn. 2024 Mar 2;9:15. doi: 10.1038/s41539-024-00219-9 (PMC10908790; doi:10.1038/s41539-024-00219-9)
Supplement: Supplementary file 2 — Reporting Summary [file 41539_2024_219_MOESM2_ESM.pdf]

nature portfolio

Corresponding author(s): Melody Wilson & Jamaal Matthews

Last updated by author(s): Dec 15, 2023

Reporting Summary

Nature Portfolio wishes to improve the reproducibility of the work that we publish. This form provides structure for consistency and transparency in reporting. For further information on Nature Portfolio policies, see our Editorial Policies and the Editorial Policy Checklist.

Statistics

For all statistical analyses, confirm that the following items are present in the figure legend, table legend, main text, or Methods section.

n/a	Confirmed	
☐	☒	The exact sample size (*n*) for each experimental group/condition, given as a discrete number and unit of measurement
☐	☒	A statement on whether measurements were taken from distinct samples or whether the same sample was measured repeatedly
☐	☒	The statistical test(s) used AND whether they are one- or two-sided<br>*Only common tests should be described solely by name; describe more complex techniques in the Methods section.*
☐	☒	A description of all covariates tested
☐	☒	A description of any assumptions or corrections, such as tests of normality and adjustment for multiple comparisons
☐	☒	A full description of the statistical parameters including central tendency (e.g. means) or other basic estimates (e.g. regression coefficient) AND variation (e.g. standard deviation) or associated estimates of uncertainty (e.g. confidence intervals)
☐	☒	For null hypothesis testing, the test statistic (e.g. F, t, r) with confidence intervals, effect sizes, degrees of freedom and P value noted<br>*Give P values as exact values whenever suitable.*
☒	☐	For Bayesian analysis, information on the choice of priors and Markov chain Monte Carlo settings
☐	☒	For hierarchical and complex designs, identification of the appropriate level for tests and full reporting of outcomes
☐	☒	Estimates of effect sizes (e.g. Cohen's *d*, Pearson's *r*), indicating how they were calculated

Our web collection on statistics for biologists contains articles on many of the points above.

Software and code

Policy information about availability of computer code

Data collection	Qualtrics was used to collect student survey responses
Data analysis	Mplus, Stata, and SPSS were used to analyze the data in this study

For manuscripts utilizing custom algorithms or software that are central to the research but not yet described in published literature, software must be made available to editors and reviewers. We strongly encourage code deposition in a community repository (e.g. GitHub). See the Nature Portfolio guidelines for submitting code & software for further information.

Data

Policy information about availability of data

All manuscripts must include a data availability statement. This statement should provide the following information, where applicable:

- Accession codes, unique identifiers, or web links for publicly available datasets
- A description of any restrictions on data availability
- For clinical datasets or third party data, please ensure that the statement adheres to our policy

The data and survey items used in the current study are available on the Open Science Framework at: https://osf.io/xbvfe/?view_only=767ee696905d4828b47f5181a89cca9d
The code used in the current study is available on the Open Science Framework at: https://osf.io/xbvfe/?view_only=767ee696905d4828b47f5181a89cca9d

April 2023

Research involving human participants, their data, or biological material

Policy information about studies with human participants or human data. See also policy information about sex, gender (identity/presentation), and sexual orientation and race, ethnicity and racism.

Reporting on sex and gender	Participants self-reported genders were used in this study.
Reporting on race, ethnicity, or other socially relevant groupings	Participants self-reported race or ethnicity were used in this study.
Population characteristics	The data for this study spanned two consecutive school years (2017-18, & 2018-19), which were the final two years (waves four and five) of a five-year study across five secondary schools in one large northeast U.S. city. According to the U.S. Census Bureau, 49.5% of the city's residents identified as Black or African American, the median income was $37,476 and 26.3% of the population lived at or below the poverty line. Schools in this city's metropolitan area retained the second highest level of Black-White school segregation in the nation.<br>The response rate for student assent and guardian consent was approximately 64%, which resulted in a sample of 285 students who self-identified as Black or African American at the beginning of the study in 2014 (M age = 12.75 years; 50.6% female). Of the students who left the schools we were able to follow some to their new schools, but the unavoidable turnover in the sample resulted in 197 and 210 participants in waves four and five respectively (T1 and T2 in this study). The total number of participants in the present analyses was 225: 41 in magnet schools and 184 in non-magnet schools.
Recruitment	Students were recruited through in-person announcements in mathematics classrooms across all five schools. Three were high schools (grades 7-12 or 9-12) and two were middle schools (grades K-8). Two of the high schools were public magnet schools that ranked within the top 20% of high schools within the state. They maintained 98% and 95% graduation rates and were approximately 33% and 22% Black American, respectively81. The third high school was a "neighborhood" school ranked within the bottom 33% of high schools within the state, maintained a 61% graduation rate, and was approximately 91% Black American. The two middle schools were also neighborhood schools, over 90% Black American, and were generally low-performing schools feeding into the predominantly Black high schools in the city.<br>Racial and mathematics beliefs were measured via survey questionnaires administered in the spring of the 2017-2018 school year (T1) and the spring of the 2018-2019 school year (T2). Math-related emotions were measured at each time point, along with perceptions of school climate (i.e., stereotyping) and classroom pedagogy (cultural and critical mathematics instruction). Student questionnaires were completed online in the school computer labs using individual computers. Typically, 10 to 25 students were surveyed at a time, and on average it took 34 minutes for students to complete the battery of survey questions. Survey questions were not randomized in their presentation. Trained undergraduate and masters research assistants under the direction of the PI and second author (a Black American man) monitored the survey administration and answered students' questions as needed but did not interact with students in any additional ways as they completed the survey items. The research team shifted in size, from 5-8 members, over the years of this study and was racially-ethnically diverse with Black, Latine, and White American research assistants.
Ethics oversight	Montclair State University IRB

Note that full information on the approval of the study protocol must also be provided in the manuscript.

Field-specific reporting

Please select the one below that is the best fit for your research. If you are not sure, read the appropriate sections before making your selection.

☐ Life sciences ☒ Behavioural & social sciences ☐ Ecological, evolutionary & environmental sciences

For a reference copy of the document with all sections, see nature.com/documents/nr-reporting-summary-flat.pdf

Behavioural & social sciences study design

All studies must disclose on these points even when the disclosure is negative.

Study description	see paper abstract, p 2.
Research sample	see "participants" section on p. 13
Sampling strategy	Convenience sampling was employed. The response rate for student assent and guardian consent was approximately 64%, which resulted in a sample of 285 students who self-identified as Black or African American at the beginning of the study in 2014 (M age = 12.75 years; 50.6% female). Of the students who left the schools we were able to follow some to their new schools, but the unavoidable turnover in the sample resulted in 197 and 210 participants in waves four and five respectively (T1 and T2 in this study). The total number of participants in the present analyses was 225: 41 in magnet schools and 184 in non-magnet schools.
Data collection	See "Data Collection" section on p. 14
Timing	All survey questionnaires administered in the spring of the 2017-2018 school year (T1) and the spring of the 2018-2019 school year

Timing	(T2).
Data exclusions	For this study we prioritized participants who self-identified as Black or African American. The study was centered around the psychological response patterns of this specific group and thus only participants who self-identified as Black or African American could help answer the research questions of this study.
Non-participation	The response rate for student assent and guardian consent was approximately 64%, which resulted in a sample of 285 students who self-identified as Black or African American at the beginning of the study in 2014 (M age = 12.75 years; 50.6% female). Of the students who left the schools we were able to follow some to their new schools, but the unavoidable turnover in the sample resulted in 197 and 210 participants in waves four and five respectively (T1 and T2 in this study). The total number of participants in the present analyses was 225: 41 in magnet schools and 184 in non-magnet schools.
Randomization	n/a there was no randomization, this was not a randomized control trial

Reporting for specific materials, systems and methods

We require information from authors about some types of materials, experimental systems and methods used in many studies. Here, indicate whether each material, system or method listed is relevant to your study. If you are not sure if a list item applies to your research, read the appropriate section before selecting a response.

Materials & experimental systems

n/a	Involved in the study	
☒	☐	Antibodies
☒	☐	Eukaryotic cell lines
☒	☐	Palaeontology and archaeology
☒	☐	Animals and other organisms
☒	☐	Clinical data
☒	☐	Dual use research of concern
☒	☐	Plants

Methods

n/a	Involved in the study	
☒	☐	ChIP-seq
☒	☐	Flow cytometry
☒	☐	MRI-based neuroimaging
